# Hematopietic Stem Cell Transplantation in Thalassemia and Related Disorders

**DOI:** 10.4084/MJHID.2009.015

**Published:** 2009-12-03

**Authors:** Emanuele Angelucci, Federica Pilo, Clara Targhetta, Martina Pettinau, Cristina Depau, Claudia Cogoni, Sara Usai, Mario Pani, Laura Dessì, Donatella Baronciani

**Affiliations:** 1 Struttura Complessa Disciplina di Ematologia e Centro Trapianto Cellule Staminali Emopoietiche “Wilma Deplano”. Ospedale Oncologico di Riferimento Regionale “Armando Businco” Cagliari; 2 Servizio Immmunoematologia e Trasfusionale, Azienda Ospedaliera “Brotzu”, Cagliari

## Abstract

The basis of allogeneic hemopoietic stem cell (HSC) transplantation in thalassemia consists in substituting the ineffective thalassemic erythropoiesis with and allogeneic effective one. This cellular replacement therapy is an efficient way to obtain a long lasting, probably permanent, clinical effective correction of the anaemia avoiding transfusion requirement and subsequent complications like iron overload. The first HSC transplant for thalassemia was performed in Seattle on Dec 2, 1981. In the early eighties transplantation procedure was limited to very few centres worldwide. Between 17 December 1981 and 31 January 2003, over 1000 consecutive patients, aged from 1 to 35 years, underwent transplantation in Pesaro. After the pioneering work by the Seattle and Pesaro groups, this therapeutic approach is now widely applied worldwide. Medical therapy of thalassemia is one of the most spectacular successes of the medical practice in the last decades. In recent years advances in knowledge of iron overload patho-physiopathology, improvement and diffusion of diagnostic capability together with the development of new effective and safe oral chelators promise to further increase success of medical therapy. Nevertheless situation is dramatically different in non-industrialized countries were the very large majority of patients live today. Transplantation technologies have improved substantially during the last years and transplantation outcome is likely to be much better today than in the ‘80s. Recent data indicated a probability of overall survival and thalassemia free survival of 97% and 89% for patients with no advanced disease and of 87% and 80% for patients with advanced disease. Thus the central role of HSC in thalassemia has now been fully established. HSC remains the only definitive curative therapy for thalassemia and other hemoblobinopathies. The development of oral chelators has not changed this position. However this has not settled the controversy on how this curative but potentially lethal treatment stands in front of medical therapy for adults and advanced disease patients. In sickle cell disease HSC transplantation currently is reserved almost exclusively for patients with clinical features that indicate a poor outcome or significant sickle-related morbidity.

## Introduction:

In easy words the rationale basis of allogeneic hemopoietic stem cell (HSC) transplantation in thalassemia consists in substituting the ineffective thalassemic erythropoiesis with and allogeneic effective one^1^. This approach is an efficient way to obtain a long lasting, probably permanent, clinical effective correction of the haemolytic anaemia^1^. Thus avoiding transfusion requirement and subsequent complications like iron overload. Allogeneic hemopoietic stem cell transplantation in genetic disease has been a cornerstone of the development of HSC transplantation^2^ and a topic of passionate debate during the past decades^3^,^4^. Although transplantation role in thalassemia is today well established this debate would be more important today in the oral chelator era and in the era of a worldwide diffuse medicine with increasing attention to the problem of the costs.

## HSC transplantation in a not malignant disease:

The issue of HSC transplant in hemo-globinopathies is conceptually more similar to solid organ transplantation than to HSC transplant in haematological malignancies. In this setting there is not a malignant clone to eradicate and a Graft versus Leukemia effect is not required. On the other hand there is not a malignant progressive disease to be included in the risk/benefit ratio analyses. Patients have not received previous chemotherapy and immunosuppression but have erythroid expansion and iron-related tissue damage. This cellular replacement therapy has been improperly defined “gene therapy today”.

All these findings must be considered in patients’ selection and transplant execution.

## Results of transplantation:

**Figure 1.**
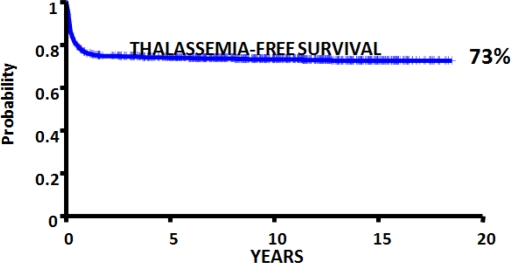
Results of HSC transplantation in 900 consecutive patients, ager 1–35 years, transplanted from an HLA identical sibling in Pesaro since December 1981.

The first HSC transplant for thalassemia was performed in Seattle on Dec 2, 1981^5^. Subsequently between 17 December 1981 and 31 January 2003, over 1000 consecutive patients, aged from 1 to 35 years, underwent transplantation in Pesaro. In fig 1 is reported the thalassemia free survival obtained in 900 consecutive patients transplanted from an HLA identical sibling. Overall, the greater than 20-year Kaplan–Meier probability of thalassemia-free survival was 73% (Figure 1)^6^.

With regard to predictors of transplant outcome, in the historical experience, patients younger than 17 years were stratified on the basis of the risk factors of hepatomegaly, a history of irregular chelation, and hepatic fibrosis^7^. With the combination of oral Busulfan 14 mg/kg and Cyclophosphamide 200 mg/kg as preparative regimen results were satisfactory for patients with no advanced disease (Class 1 and Class 2) while were unacceptable for patients with advanced disease (Class 3)^7^. For the first two category of patients this conditioning regimen has remained unmodified since 1985^8^. For the discouraging results with this conditioning regimen in patients with advanced disease, after 1990 class 3 patients underwent transplantation based on protocols that included lower dosages of cyclophosphamide (120–160 mg/kg)^9^. Transplantation in class 3 children, with this regimen was characterized by a 30% risk of thalassemia recurrence^9^. In contrast, in adults with the same dose of cyclophosphamide, thalassemia recurrence was only 4%, but there was a 35% transplant-related mortality^10^. These historical Pesaro results are reported in Table 1. Because class 3 children had the problem of thalassemia recurrence whereas adults had the problem of transplant-related mortality a new preparative regimen was introduced for class 3 patients with the aim of increasing the rate of sustained engraftment while decreasing transplant-related mortality. This regimen was characterized by a 30-day preconditioning period designed to produce erythroid cytoreduction and immune-suppression before a conditioning regimen using a reduced dose of cyclophosphamide. In a pilot study of 33 consecutive class 3 thalassemia children, the 5-year Kaplan–Meier probability of survival and disease free survival was 93% and 85%, respectively^11^.

## Application of transplantation world-wide:

**Figure 2.**
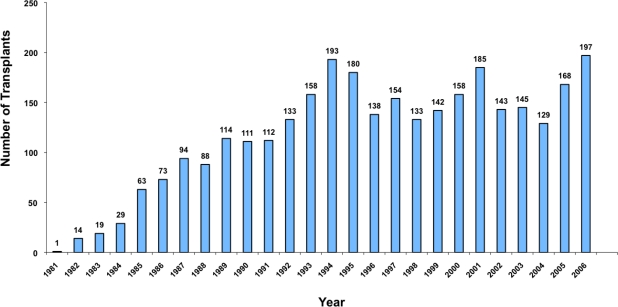
Numbers of HSC transplants performed for thalassemia in centers of the European Registry for Blood and Marrow Transplantation (EBMT). Unpublished data from the EBMT Hemoglobinopathy Registry

**Figure 3.**
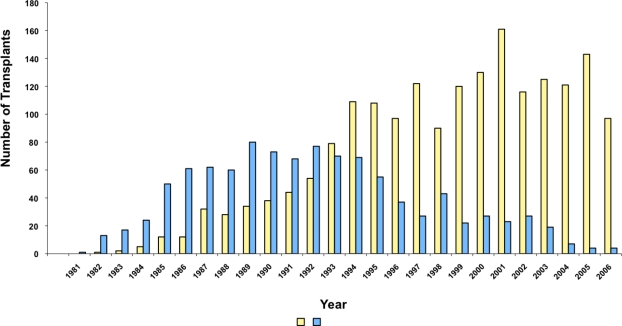
Numbers of HSC transplants performed for thalassemia through the years in centres of the European Registry for Blood and Marrow Transplantation (EBMT). The blue bars indicate transplants performed in Pesaro. The yellow bars indicate transplants performed in all the other centres. Unpublished data from the EBMT Hemoglobinopathy Registry

After have been pioneered by the Seattle group^5^, the Pesaro group^4^,^7^,^8^,^12^–^15^ and the Pescara group^16^ this therapeutic approach is now widely applied worldwide^6^. In the early eighties transplantation procedure was limited to very few centres worldwide. The European Group for Blood and Marrow Transplantation has recently established the hemoglobinopathy registry and it is possible today to a have detailed epidemiology data on over 3,000 patients registered. After the early ninety a constant numbers of transplants raging between 133 and 197 per year have been registered with a constant behaviour during the years (Figure 2)^6^. The pioneering role of the Pesaro group is today clear (Figure 3) and the wide dissemination of this practice after 1993 is the best demonstration of its success.

## Transplantation versus medical therapy:

Medical therapy of thalassemia is one of the most spectacular successes of the medical practice in the last decades. Thalassemia has been transformed from an infant lethal disease in a chronic disease permitting prolonged survival. An Italian retrospective study^17^ clearly demonstrated which has been the medical progress in this setting comparing survival of patients born in different decades. Nevertheless situation is dramatically different in non-industrialized countries were the very large majority of patients live today.

In recent years advances in knowledge of iron overload patho-physiopathology, improvement and diffusion of diagnostic capability together with the development of new effective and safe oral chelators^18^,^19^ promise to further increase success of medical therapy^20^. Particularly today well-studied oral chelators offer a concrete alternative to deferoxamine infusion therapy.

The problem of the choice between transplantation versus medical therapy is a kind of question, which cannot be resolved, for several, obvious, reasons by a prospective randomized clinical trial. In absence of such data the choice should be done on analyses of large prospective (transplant) and retrospective (medical therapy) phase II trials and individual preference. In this kind of clinical question several aspects must be kept in consideration making the decisional process different in individual cases: age, clinical status, capability and compliance to adhere a correct transfusional-chelation regimen and resources. In case of children the dilemma dramatically relies on parents shoulders.

## Resources availability:

Even if not properly medical issue resources availability is a fundamental issue. A modern complete transfusion – chelation therapy is a high technology therapy requiring important expertise. Even not considering complication, medical therapy is very expensive clearly not widely available worldwide^21^. Even transplantation is a high technology therapy but the diffusion of transplant centres is greatly increased today^22^. It has been calculated that in term of resources availability and utilization HSC transplantation corresponds to approximately 3 years of transfusion- chelation therapy with deferoxamine not including complications costs.

In large part of the world, were today lives the large part of thalassemia patients, the choice between transplant and medical therapy cannot consider just reported literature but resources availability also.

## Alternative source of stem cells:

The large majority of transplant in thalassemia has been performed using bone marrow derived stem cells. In the EBMT survey more than 80% of the transplants have been performed using bone marrow derided cells. Even in the most recent years, although less pronounced, bone marrow derived stem cells continue to be the preferred source of stem cells for transplantation in thalassemia.

In 1998 Franco Locatelli first proposed the use of identical sibling derived hemopoietic stem cells for transplantation in hemoglobinopathies^27^. Since that time use of cord blood derived hemopoietic stem cell has much expanded^28^ with outstanding results in the field of hemo-globinopathies particularly regards a very low mortality rate.

## Alternative donor:

As very well known in transplantation only approximately 1/3 of patients has an HLA identical sibling inside the family^23^. Use of alternative donors includes matched unrelated donor, mismatched donors. The first approach has been applied by a multicentre Italian study and demonstrated satisfactory results^24^. However the limited number of cases reported (< 100 in more than 10 years) and the limitation in donor selection makes this procedure be reserved to specialized centres and controlled trials.

Unrelated cord blood transplantation is standard practice today for HSC therapy in malignancies. Tolerance of 1–2 HLA antigens mismatch, fast availability and low incidence of graft versus host disease makes this option very attractive for not malignant diseases including thalassemia. Preliminary experience is promising but limited (36 reported cases with a 77% overall survival and 65% thalassemia free survival)^25^ and, therefore, should be considered a experimental approach. Mismatched donor transplantation has been issue of a very limited number of trials^26^. Because of the experimental role of this procedure and the alternative of medical therapy available today this procedure should be not recommended unless in very selected clinical cases lacking an HLA compatible donor in which medical therapy has been formally documented to be not feasible.

## Alternative conditioning regimens:

Be-cause of the lack of a malignant clone to eradicate and the need to reduce toxicity non-ablative transplantation would have theoretically a role in thalassemia and hemoglobinopathies. However attempts performed in this direction failed. This was recently reported in two limited experiences^29^,^30^. Overall eleven patients with Thalassemia or Sickle cell Disease received a non-myeloablative regimen before HSC transplant. As expected transplant related mortality was very limited but only one patient had a sustained engraftment. This was probably due to expanded erythroid clone and the immunological situation demonstrating that a stable allogeneic engraftment is difficult to obtain in hemoglobinopathies and additional immunological tools would be necessary. However in the setting of a not-malignant disease the addiction of post transplant donor lymphocyte infusion does not appears to a rational approach in terms of risk/benefit ratio.

## Mixed chimerism:

In the setting of HSC transplantation mixed chimerism is defined as the concurrent presence of donor and host hemopoietic cells. Mixed chimerism is commonly reported after HSC transplant for thalassemia after a myeloablative-conditioning regimen. In a prospective study by Andreani in over 300 consecutive thalassemia patients^31^ early mixed chimerism was demonstrated in 28% of the patients. Of them only approximately one fourth (11% of the surviving patients) confirmed mixed chimerism in a prolonged follow up extending over the second year after transplant (persistent chimerism). In a recent study from Pavia this behaviour has not been confirmed in the setting of cord blood transplantation^28^.

Very interesting and surprising in those patients who presented persistent mixed chimerism a partial engraftment, up to the 20% of marrow cells, was still able to maintain a normal hemoglobin level avoiding blood transfusions. In this population, no sign of increasing iron overload or other clinical complication of thalassemia or thalassemia intermedia were detectable, thus obtaining a complete clinical control of the disease.

Regardless of mechanism correction of anemia by relatively low amount of mixed chimerism provide the rationale for development of minimally–ablative conditioning regimens to obtain a partial engraftment and at the same time abolish or drastically decrease transplant related mortality. The evidence that a partial engraftment of a normal erythropoiesis provide clinical control of the disease could also be the basis for a gene therapy program (when safely available) since eradication of the thalassemic marrow is not necessary to clinical control of the disease.

## Transplantation for thalassemia in the era of oral chelators:

The central role of HSC in thalassemia has now been fully established. Transplantation remains the only definitive curative therapy for thalassemia and other hemoglobinopathies. The development of oral chelators has not changed this position. However this has not settled the controversy on how this curative but potentially lethal treatment stands in front of medical therapy for adults and advanced disease patients. Transplantation technologies have improved substantially during the last years and transplantation outcome is likely to be much better today than in the ‘80s. Recent data indicated a probability of overall survival and thalassemia free survival of 97% and 89% for patients with no advanced disease and of 87% and 80% for patients with advanced disease 32. Similar data are coming from the EBMT survey on over 1000 patients transplanted in the EBMT centres during the last 10 years with a over 90% overall survival at 5 years.

## Transplantation for Sickle Cell Disease:

Similar to β thalassemia major, the objective of HSC transplantation for sickle cell disease is to substitute recipient erythropoiesis, or to reduce its clinical impact, by the expression of donor β-globin chains. The clinical benefit of this cellular replacement is the elimination, or noteworthy amelioration, of the clinical complications caused by polymerized sickle hemoglobin. Since the initial clinical trials of HSC transplantation for sickle cell disease it was clear that the replacement of donor for sickle erythropoiesis might abolish not only the hematological manifestations of the underlying disorder, but also stabilize and even decrease the organ damage caused by previous recurrent vessel occlusion and haemolysis^33^–^36^. As in thalassemia there is usefulness in assigning a risk-based approach to applying transplantation for sickle cell disease. Unfortunately, defining risk characteristics is difficult in the absence of prospective trials. Nonetheless, the appropriate broader application of HSC transplantation hinges on two important objectives.

1. To identify those patients who have the greatest risk of developing sickle-related complications and who are most likely to benefit from hematopoietic cell transplantation.
2. To reduce transplant-related complications by minimizing the short- and long-term toxicities of HSC transplantation.

Unlike β-thalassemia major, where the genotype directs a reasonably reliable phenotype in the vast majority of cases, the clinical expression of sickle cell anemia is quite variable and difficult to predict based upon the hemoglobin genotype alone. Thus, in standard practice, HSC transplantation for sickle cell disease currently is reserved almost exclusively for patients with clinical features that indicate a poor outcome or significant sickle-related morbidity, in part due to the toxicity of this intensive therapy^37^. These clinical indications are listed in Table 2.

These criteria have been developed and applied almost exclusively on children, for whom the risk–benefit ratio is most advantageous in terms of years-of-life gained. Less certain is how to apply inclusion criteria to adults with sickle cell anemia, in whom the experience of transplantation is limited, but for whom the risk of significant transplantation-related toxicity remains substantial. For all patients, clinicians must carefully weigh therapeutic alternatives to hematopoietic cell transplantation, with particular attention to safety, efficacy, availability, and the cost of intervention^38^–^40^.

The worldwide experience of conventional myeloablative hematopoietic cell transplantation for sickle cell disease^34^–^36^,^41^–^44^ is summarized in Table 3. In the collective experiences of these studies, HSC transplantation moved from an experimental intervention reserved for severely affected patients, to one in which younger children with early signs of sickle-related morbidity are included. The results of transplantation were best when performed in children with sickle cell disease who had HLA-identical sibling donors. Even though many children who received allografts had significant sickle-related complications such as stroke and recurrent episodes of acute chest syndrome, the disease-free survival rate was 80% to 85% quite similar in the different experiences; however, 5%–10% of patients died of complications related to transplantation, with GVHD and its treatment the leading cause of death

As in thalassemia major, the observation of donor–host hematopoietic chimerism after conventional myeloablative hematopoietic cell transplantation has lent substantial support to the notion that persistence of even a fraction of normal engrafted erythropoiesis might elicit a curative clinical effect^31^,^44^ leading to the same potentiality for a gene therapy approach as described for thalassemia.

## Conclusion:

HSC transplantation in hemoglobinopathies has today a well-recognized place as therapeutic option for these inherited diseases. As elsewhere described in this journal, these diseases have a tremendous impact with hundred of thousands of patients (mostly children) affected worldwide of whom few have the transplant option. As above described the transplant decision is much different in the two diseases requiring deep knowledge of the diseases and at the same time of the transplant possibility and limitation. Any effort should be done to further decrease transplant related mortality and to extend transplantation option to patients so far lacking an HLA identical donor and to those without modern healthcare resources.

## Figures and Tables

**Table 1.** Results of transplantation. Historical results from Pesaro experience during the eighties and nineties.

	**Regimen**	**Overall Survival**	**Thalassemia Free Survival**
Class 1	Bu 14 – Cy 200	93 %	90%
Class 2	Bu 14 – Cy 200	87%	84%
Class 3	Bu 14 – Cy 120 -160	79%	58%
Adults	Bu 14 – Cy 120 -160	66%	62%

**Table 2.** Indications of Hematopoietic Cell Transplantation for Sickle Cell Disease

One or more of the following complications: Stroke or central nervous system event lasting > 24h. Impaired neuropsychological function with abnormal cerebral MRI and angiography Recurrent acute chest syndrome Stage I or II sickle lung disease Recurrent vaso-occlusive painful episodes or recurrent priapism Sickle nephropathy (glomerular filtration rate 30% – 50% of predicted normal values)
Other indications to be considered: Abnormal transcranial Doppler, pulmonary hypertension, silent cerebral infarction.

**Table 3:** Worldwide results obtained by Myeloablative HSC transplantation in Sickle Cell Disease.

	**US Collaborative**	**French**	**Belgian**	**CIBMTR**
Patients	59	87	50	67
Median age	9.9 (3.3 – 15.9)	9.5 (2–22)	7.5 (0.9 – 23)	10 (2–27)
Disease Recurrence.	8.5%	8%	10%	13%
Deaths	7%	7%	4%	4.5%
Event Free survival	85%	85%	86%	82%
Patients with Chronic GvHD	12%	12.6%	20%	22%
